# Measuring the Electron’s Charge and the Fine-Structure Constant by Counting Electrons on a Capacitor

**DOI:** 10.6028/jres.097.010

**Published:** 1992

**Authors:** E. R. Williams, Ruby N. Ghosh, John M. Martinis

**Affiliations:** National Institute of Standards and Technology, Gaithersburg, MD 20899; National Institute of Standards and Technology, Boulder, CO 80303

**Keywords:** calculable capacitor, coulomb blockade, electron charge, electron counting, fine-structure constant, single electron tunneling

## Abstract

The charge of the electron can be determined by simply placing a known number of electrons on one electrode of a capacitor and measuring the voltage, *V*_s_, across the capacitor. If *V*_s_ is measured in terms of the Josephson volt and the capacitor is measured in SI units then the fine-structure constant is the quantity determined. Recent developments involving single electron tunneling, SET, have shown bow to count the electrons as well as how to make an electrometer with sufficient sensitivity to measure the charge.

## 1. Introduction

The recent development of single-electron devices [1,2,3,4,5] has made possible a new and very precise technique to measure the charge of an electron. These devices are based on metal/insulator/metal tunnel junctions whose current-voltage (*I–V*) characteristics are determined by individual electron tunneling events. If such a device is used to place *n* electrons on a capacitor of capacitance *C*_s_ and the resulting voltage *V*_s_ is measured, the electron charge *e* can be determined in terms of this voltage and capacitance, *ne = V*_s_*C*_s_. In practice, *V*_s_ is most precisely determined in terms of the voltage generated by a Josephson junction and thus the experiment will not be a determination of *e* in SI units, but a measurement of *e* in terms of the Josephson volt [6]. In this paper we show that such a measurement will soon be possible and that an accurate result would make a useful contribution to the field of fundamental physical constants.

### 1.1 Relating the Fine-Structure Constant and the Electric Charge

First, we address how this measurement relates to the fine-structure constant, *a*. By relating the measured voltage to a Josephson voltage and the capacitance to SI capacitance as measured in a calculable capacitor experiment [7], we can calculate the fine-structure constant from the following simple equations:

$$V_{s}=\frac{ne}{C_{s}}=jf_{i}\frac{h}{2e}$$ (1)

where *f_i_* is the frequency producing the Josephson steps and *j* is an integer associated with the Josephson effect. (Note that in a Josephson array *j* is the sum of all the integer steps of each junction.) Solving Eq. (1) for *e*^2^/*h*, α is given by

$$\alpha =\frac{\mu_{0}ce^{2}}{2h}=\frac{j\mu_{0}cf_{i}C_{s}}{4n}$$ (2)

where *μ*_0_ is the permeability of vacuum and *C*_s_ must be measured in SI units. Specifically, the capacitance must be related to the calculable capacitor experiment. In a calculable capacitor experiment a change of capacitance of 0.5 pF can be measured in terms of the meter with an accuracy of 0.014 ppm [7] (1 ppm = l × 10^−6^; throughout, all uncertainties are one standard deviation estimates). This capacitance can then be related with accuracies near the 0.01 ppm level to a 10 pF capacitor that is stable and transportable.

If this experiment to measure *e* in terms of the Josephson volt and the calculable capacitor can be realized with high accuracy, it will provide a new path to *α*. This new approach is similar to the measurement of *α* via the quantum Hall effect [8]. Although both experiments require a connection to the calculable capacitor, this new method is much more direct. The 0.5 pF capacitance change used in most calculable capacitor experiments is a good match to the size of the capacitance that might be used to determine *e*; only one or two precision ratio transformer bridges need be involved in the calibration of *C*_s_. By contrast, in the quantum Hall case a chain of calibrations might involve these intermediate standards: 0.5 pF, 10 pF, 100 pF, 1000 pF, 100 kΩ, 10 kΩ, 1 kΩ, and 6453.2 Ω. The most accurate value of *α* determined from the calculable capacitor and quantum Hall effect has an uncertainty of 0.024 ppm [7,9] with 0.014 ppm coming from the calculable capacitor and the rest from the chain of intermediate standards. An alternate determination of *α* using the proton gyromagnetic ratio in H_2_O and the quantum Hall effect but not the calculable capacitor, has an uncertainty of 0.037 ppm [9]. However, these two results differ from each other by (0.10 ± 0.043) ppm. A value of *α* determined from the anomalous magnetic moment of the electron and quantum electrodynamic theory (QED) has an uncertainty of 0.007 ppm [10] and its value lies between the two non-QED values. Therefore, the unexplained 0.10 ppm difference in the non-QED values limits the accuracy to which QED theory is tested. Any measurement of *α* by this new SET method at the 0.1 ppm level or better would be helpful in the field of fundamental constants [8].

### 1.2 SET Devices

K. K. Likharev and his colleagues [1] have long been proponents of the application of Coulomb blockade effects arising from the discreet charge of electrons to realize a precise current source. Their pioneering ideas and experiments paved the way for much of the recent progress. In the past few years, new devices employing metal/insulator/metal tunnel junctions have been demonstrated which could allow a precise measurement of the electron charge *e* and thus *α*. These include an electrometer [2], observation of single electron tunneling oscillations [3], a “turnstile” current source [4], and a “pump” current source [5]. A brief sketch of single-electron tunneling is presented to describe the operation of the electrometer and pump.

Consider a normal-metal tunnel junction biased by a current source and having a capacitance *C*. The change in energy of the junction after an electron tunnels through the barrier is *ΔE* = *eQ/C − e*^2^*/*2*C*, where *Q* is the charge across the junction and *e*^2^*/*2*C* is the Coulomb energy cost of the tunneling event. If the tunneling resistance of the junction is greater than the quantum unit of resistance, *R_t_* ⪢ *h/e*^2^, and thermal fluctuations do not mask the charging energy, *kT* ⪡ *e*^2^*/*2*C*, then a Coulomb blockade appears in the junction *I–V* characteristic where the tunneling probability is greatly reduced for |*V*| *< e*/2*C*. At 50 mK, and using 1 fF for the junction capacitance, one finds (*kT*)/(*e*^2^*/*2*C*) ~ l/20, hence temperature effects are small but non-negligible. The single-electron devices exploit this Coulomb blockade of the tunneling current.

The Fulton-Dolan (SET) electrometer [2] is shown schematically in Fig. la. Aside from tunneling events, the electrode a between the two junctions and the gate capacitor Co is an “island” electrically isolated from the circuit. The electrometer provides a very high impedance technique to measure the potential *U*. At a constant bias *V*, the device conductance as a function of gate voltage is periodic with the period *ΔU = e/C*_0_. The bias voltages *V* and *U* are chosen to maximize the sensitivity of the electrometer so that the device current is linearly proportional to small changes of the potential *U*. Such electrometers have demonstrated charge sensitivity of 1.5 × 10^−4^ *e*/Hz^1/2^ at a frequency between 2 and 200 Hz [11].

Convincing experimental evidence of the SET oscillations producing the controlled transfer of electrons with the relation *I = ef* was reported by Delsing et al. [3]. Geerligs et al, [4] were the first to demonstrate flat plateaus and observe a current with accuracies of better than 1% using the turnstile device. However, certain properties of the pump device suggest that it will be more precise in counting electrons and we therefore sketch its operation.

**Fig. 1a:**
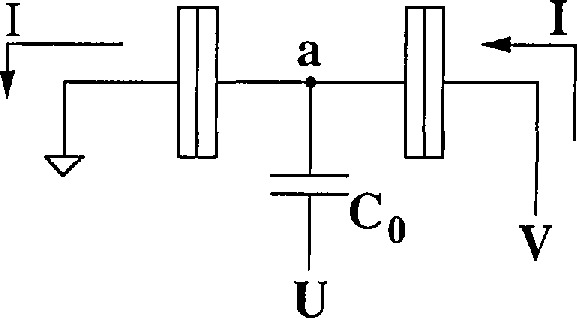
This circuit shows an electrometer formed by two tunnel junctions each having a capacitance *C*. The input is potential *U* coupled to the isolated island a through *C*_o_.

An electron pump [5] (see Fig. 1b) is a device similar to the electrometer, in which a single electron tunnels sequentially through the “islands” labeled b and c. For a given voltage *V*, a controlled number of electrons can be transferred in either direction by appropriately cycling the gate voltages *U*_1_ and *U*_2_ at the frequency *f*. The pump also delivers an electron current at the rate *I = ef*. The direction of the current can be reversed simply by reversing the phase of *U*_1_ and *U*_2_. *A* pump based on four or five tunnel junctions operating at 1–10 MHz has been theoretically predicated to be able to deliver a known current with errors below the 1 ppm level [12,13].

The currents produced by these pumps are only about 10^−12^ A and thus precision measurements will be a challenge. Direct measurement by sensing the voltage across a high resistance is a problem because it is difficult for metrologists to accurately calibrate the required high resistances. Capacitors, on the other hand, have already been accurately calibrated in a range that will be useful in the following experiment.

## 2. Experimental Configurations [14]

### 2.1 Measurements of the Electric Charge *e*

Figure 2a shows one possible configuration that incorporates these new SET devices to measure the electronic charge. A standard capacitor *C*_s_ is connected to a current source (pump) and also to the input of a Fulton-Dolan electrometer via a coupling capacitor *C*_c_, forming an isolated island a. Using the electrometer as a null detector a voltage *V*_s_ is applied across *C*_s_ to maintain the island a at a fixed potential (near ground), while pumping electrons onto or off of the island. The stray capacitance to ground, *C*_g_, limits the sensitivity of the electrometer to *V*_s_. The voltage, *V*_s_, on the capacitor is adjusted so that the electrometer input is always at the same potential (near ground). Thus the current from the pump serves as the charging current of the capacitor as the voltage is ramped up. A specific example is illustrated in Fig. 2b. With the pump operating at 6.2 MHz for 10 s, the voltage across a 1 pF capacitor standard will charge to 10 V at a linear rate in order to keep the electrometer input constant. We stop the pump and measure the 10 V signal as well as any deviation of the electrometer from null. Reversing the pump for 20 s then brings the voltage to −10 V.

**Fig. 3:**
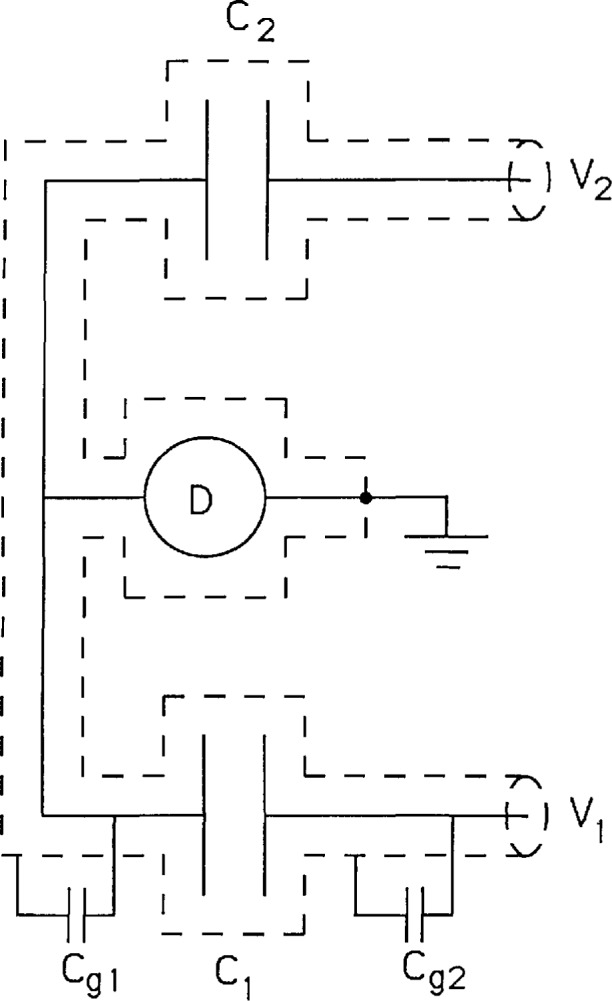
Circuit showing half of a bridge used to compare capacitors C_1_ and C_2_. The potentials *V*_1_ and *V*_2_ are usually supplied by a precision transformer. The effect of stray capacitances *C*_g1_ and *C*_g2_ are discussed in the text.

In order to use a capacitor as a standard it must have a well defined capacitance; it thus is necessary to understand how precision capacitors are measured and under what circumstances they are well defined. Figure 3 shows the essential features of one side of a standard bridge that is used to compare capacitors *C*_1_ and *C*_2_. The dashed lines represent the grounded shield that prevents the potentials *V*_1_ and *V*_2_ from affecting the detector except through their respective capacitances. This shielding is critical to making precise capacitance comparisons. The bridge is balanced by adjusting the potentials *V*_1_/*V*_2_ *= C*_2_*/C*_1_. *C*_g1_ and *C*_g2_ represent the stray capacitance to ground. The bridge balance is not affected by *C*_g1_ because there is no voltage across *C*_g1_ when the bridge is balanced. However, *C*_g1_ does affect the detector sensitivity so it should be kept small. The capacitance *C*_g2_ does not affect the balance as long as the impedance from the source to *C*_1_ is small compared to the impedance of *C*_g2_. Present day precision 10 pF capacitance standards with *C*_g1_ = 200 pF are compared at the 0.01 ppm level at a frequency *ω* = 10^4^ s^−1^ (1592 Hz) using a room temperature detector with a noise figure of 20 *e/*Hz^1/2^. A SET electrometer should have similar noise performance at the same frequency and capacitance. Further improvements in the sensitivity of the SET electrometer are expected when measuring smaller capacitances.

The key to this experiment to measure *e* using a capacitor is to replace the detector in Fig. 3 with a Fulton-Dolan electrometer as shown in Fig. 2a. By placing the electrometer at 20 mK near the source of charge, we anticipate fewer problems from leakage and less stray capacitance to ground. In addition, we expect to gain from the impressive sensitivity of this device, reduce the leakage dramatically, and still make a well defined capacitor that can be calibrated *in situ*.

Because of the requirement to shield the standard capacitor, it is easier initially to have this capacitor on a different chip from the electrometer and pump. As multilayer technology becomes incorporated into the junction fabrication process this capacitor could be placed on the same chip. Having it on a separate chip leads to some practical limits in the optimum choice for *C*_s_. The sensitivity of the electrometer is approximately given by *C*_c_/(*C*_s_ + *C*_g_), where *C*_g_ represents all other capacitances to ground. This two chip arrangement will increase *C*_g_, but we expect to keep it near 1 pF. Choosing *C*_s_ to be small both increases *V*_s_ and maximizes the electrometer sensitivity. A 1 pF capacitance for *C*_s_ constitutes a practical compromise.

Calibration of the capacitance requires making a connection to room temperature. However, the Fulton-Dolan electrometer could still be used by means of the calibration input switch shown in Fig. 2. Note that care must be taken to keep the capacitance from the island to ground low.

### 2.2 Dual Pump Bridge

**Fig. 4:**
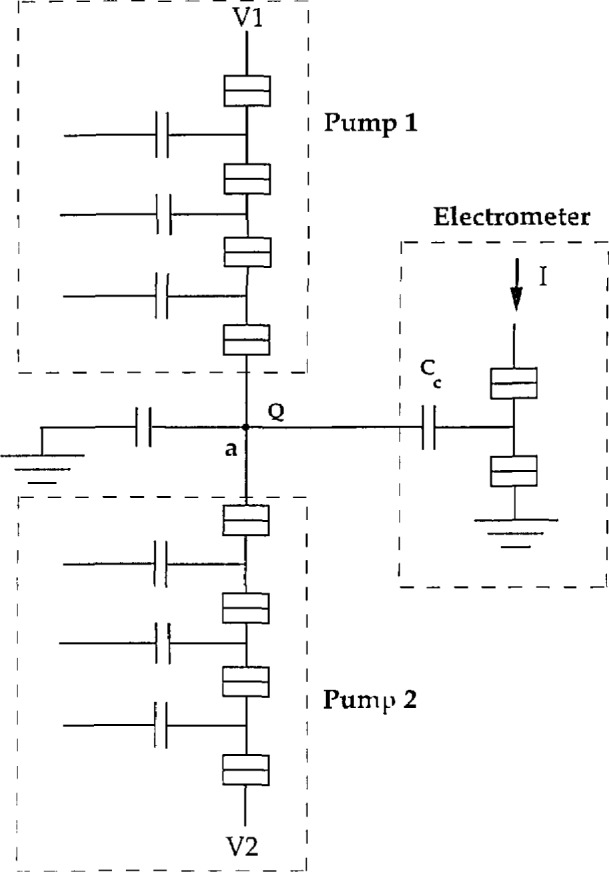
A bridge circuit used to compare two pump circuits. The electrometer detects any accumulation of charge on island a.

Another interesting experiment (see Fig. 4) is to have two current sources (drawn as two, four-junction “pumps” in Fig. 4) feeding into an island a with a total capacitance of about 10 fF. The charge state of island a is then measured with an electrometer. This geometry will not measure *e* but will allow the very precise comparison of two current sources. Any difference in current from the two sources will show up as an accumulation of the island charge. The island capacitance is chosen to be large enough to greatly reduce the interaction of the two pumps but small enough to still have single charge resolution. Because the error is detected as an integrated charge on the island, only a short time is required to obtain extremely high precision. It would also be interesting to compare the error rate of a “pump” and a “turnstile.”

## 3. Future Prospects

A new precision technique to measure the fine-structure constant via Eqs. (1) and (2) has been described. At present SET experiments have been used to determine currents only at the 0.1 to 1.0% level, hence it is risky to predict the ultimate accuracy of the technique. Based on theoretical predictions [12,13] we expect that the number of electrons could be measured with 1 ppm accuracy in a four junction pump. A five junction pump should be more accurate still. At present the electrical standards needed to support this measurement do not appear to limit its accuracy. For example, capacitance standards of 10 pF are compared between national laboratories near the 0.02 ppm level and 10 V Zener voltage references are also compared with 0.02 ppm accuracy. In the first experiments these accuracies will likely not be reached, but much physics will be learned about SET devices as we make another accurate determination of *α*.

## Figures and Tables

**Fig. lb:**
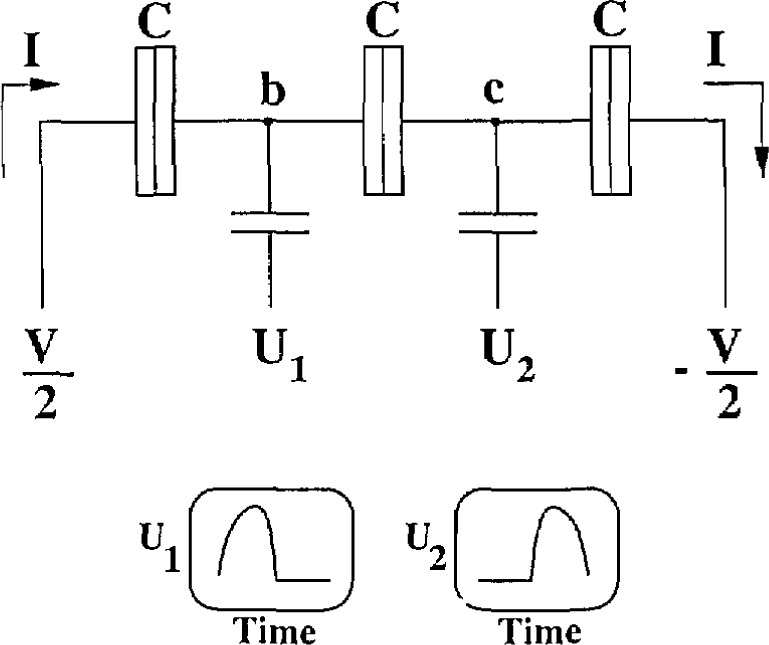
This circuit shows a three junction pump that pumps one electron per cycle of the wave-forms shown. The electron is first pumped to island b by potential *U*_1_ then to c by *U*_2_.

**Fig. 2a:**
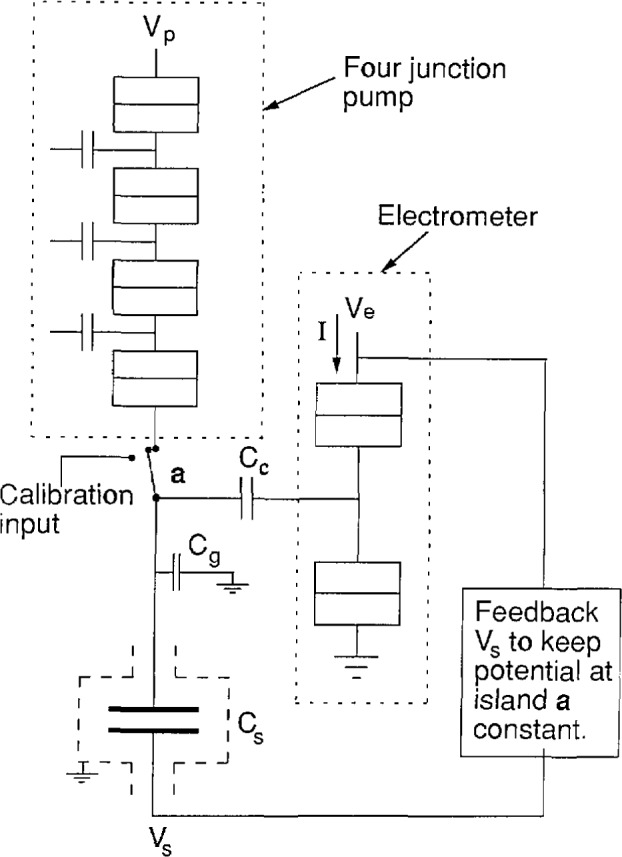
Proposed circuit for measuring *α*. The voltage *V*_s_ required to keep the potential of the island a constant when *n* electrons are pumped onto the island can be used to measure the electron charge in terms of *C*_s_, and *V*_s_. The electrometer monitors the potential at a.

**Fig. 2b:**
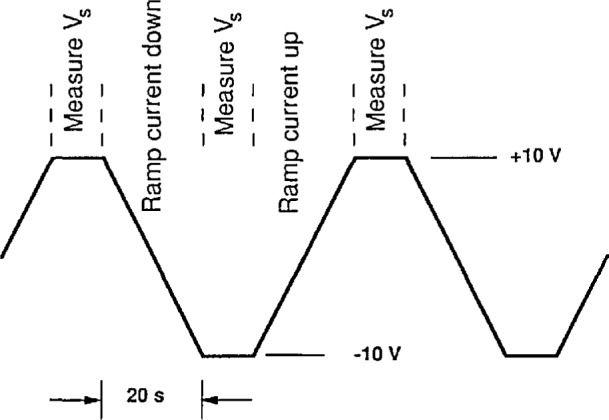
The measurement sequence for measuring *α*, showing the potential *V*_s_ as a function of time.
